# Deforestation Increases the Risk of Scrub Typhus in Korea

**DOI:** 10.3390/ijerph16091518

**Published:** 2019-04-29

**Authors:** Kyung-Duk Min, Ju-Yeun Lee, Yeonghwa So, Sung-il Cho

**Affiliations:** 1 Institute of Health and Environment, Seoul National University, 1 Gwanak-ro, Gwanak-gu, Seoul 08826, Korea; kdmin11@hotmail.com; 2 Department of Public Health Science, Graduate School of Public Health, Seoul National University, 1 Gwanak-ro, Gwanak-gu, Seoul 08826, Korea; inezkorea@snu.ac.kr (J.-Y.L.); anadagio@snu.ac.kr (Y.S.)

**Keywords:** scrub typhus, spatiotemporal models, deforestation, One Health

## Abstract

*Background*: Scrub typhus is an important public health issue in Korea. Risk factors for scrub typhus include both individual-level factors and environmental drivers, and some are related to the increased density of vector mites and rodents, the natural hosts of the mites. In this regard, deforestation is a potential risk factor, because the deforestation-induced secondary growth of scrub vegetation may increase the densities of mites and rodents. To examine this hypothesis, this study investigated the association between scrub typhus and deforestation. *Methods*: We acquired district-level data for 2006–2017, including the number of cases of scrub typhus reported annually, deforestation level, and other covariates. Deforestation was assessed using preprocessed remote-sensing satellite data. Bayesian regression models, including Poisson, negative binomial, zero-inflated Poisson, and zero-inflated negative binomial models, were examined, and spatial autocorrelation was considered in hierarchical models. A sensitivity analysis was conducted using different accumulation periods for the deforestation level to examine the robustness of the association. *Results*: The final models showed a significant association between deforestation and the incidence of scrub typhus (relative risk = 1.20, 95% credible interval = 1.15–1.24). The sensitivity analysis gave consistent results, and a potential long-term effect of deforestation for up to 5 years was shown. *Conclusion*: The results support the potential public health benefits of forest conservation by suppressing the risk of scrub typhus, implying the need for strong engagement of public health sectors in conservation issues from a One Health perspective.

## 1. Introduction

**Figure 1:**
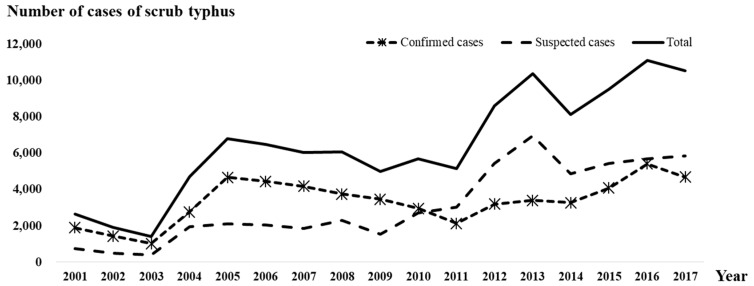
The number of cases of scrub typhus reported annually in Korea.

Scrub typhus is a mite-borne disease caused by Orientia tsutsugamushi that is transmitted mainly by trombiculid mites, such as Leptotrombidium pallidum and Leptotrombidium scutellare, in Korea [1]. In the transmission dynamics, rodents play an important role, because the mites are mainly parasitic on rodents and become infected by feeding on infected rodents. The burden of the disease is higher than that of other vector-borne diseases in South Korea [2]. According to the national passive surveillance system established by the Korea Centers for Disease Control and Prevention (KCDC), 110,070 scrub typhus cases were reported between 2001 and 2017 (56,677 confirmed cases and 53,393 suspected cases), with approximately 10,000 cases reported annually between 2013 and 2017 (Figure 1). The increasing vigilance of the surveillance system might have affected the upward trend to some degree. However, the expansion of the geographic distribution of vectors and their increasing density associated with climate change is suspected to be a major contributor to the trend [3]. Various interventions have been implemented every fall, the peak season for scrub typhus in Korea, including public awareness campaigns, especially in endemic rural regions. However, the number of cases has not decreased as expected. Rather, concerns have been growing due to recent evidence of the disease’s urbanization [4].

Previous studies identified both individual- and environmental-level risk factors associated with the transmission dynamics of scrub typhus [5]. Older age was reported to be associated with a higher incidence [1,6,7]. Agriculture-related work activities [8,9] and awareness of the disease among farmers [8] are also significant risk factors, related to the exposure level. Climate factors [10,11,12], including temperature, precipitation, the amount of sunshine, atmospheric pressure, and other environmental factors such as latitude [13] have been investigated as possible drivers or effect modifiers of the increasing scrub typhus incidence, by affecting mite or rodent densities [12].

Deforestation, defined as land cover change from forest to non-forest regions, could be another risk factor. The deforestation-induced secondary growth of scrub vegetation would provide a suitable environment for rodents, which are the natural hosts of vector mites [14], and may increase the density of mites. To the best of our knowledge, there is no empirical evidence of an association between deforestation and scrub typhus. However, significant associations with other vector-borne diseases, such as malaria, have been found [15,16,17,18], implying that similar associations could be found in scrub typhus.

This study investigated the effects of deforestation on the incidence of scrub typhus. Since deforestation, as the major explanatory variable, can be evaluated at the aggregate level, we used an ecological study design in which the analysis units were 250 districts, a second-level administrative region in Korea. The outcome variable and other covariates were measured at the aggregate level in each district. The study period was 12 years from 2006 to 2017, based on data availability. 

## 2. Materials and Methods

### 2.1. Data Acquisition and Preprocessing

This study used data from multiple sources to examine the hypothesis, including the number of scrub typhus cases reported annually by each district, the annual deforestation level, and other covariates that are considered to be associated with scrub typhus (Table 1).

The numbers of scrub typhus cases reported annually by each district were obtained from the infectious disease portal [2] of the KCDC. According to KCDC notification guidelines [23], the reported cases included both suspected and confirmed cases. The suspected case was defined as a patient who presented clinical signs of scrub typhus (acute febrile illness, lymphadenopathy and/or skin eschar) and showed epidemiological evidence. The confirmed case was defined as a patient who met both clinical and laboratory confirmation (equal or more than 4-fold increase in the antibody titer in paired serum sample, antigen detection in blood or skin eschar by polymerase chain reaction, equal or higher single antibody titer measured at 1:256 (IgG) or 1:16 (IgM) by indirect immunofluorescent antibody (IFA) test). Although KCDC provided the number of confirmed and suspected cases separately at the national level, the categorized data was not accessible at the district level. In this regard, the outcome measurement in this study included both confirmed and suspected cases. While the reported cases for each district were used as an outcome variable, we also included the expected numbers of cases in each district in the analysis as an offset for sex and age standardization.

Forest cover and annual deforestation levels were acquired from the website provided by Hansen et al. [19], and derived from satellite images (Global Forest Change, http://earthenginepartners.appspot.com/science-2013-global-forest). Using satellite images obtained during the growing seasons between 2000 and 2017, Hansen et al. used a classification algorithm that categorized the captured terrestrial surface into forest and non-forest regions, and assessed forest presence in 2000 and annual deforestation levels from 2001 to 2017 [19]. The authors provided the two variables as raster type data, which were composed of pixels containing values. Each pixel measured approximately 30 × 30 m for both variables. The forest cover variable was provided for the year 2000 and ranged from 0% to 100%, indicating the probability of forest being present within each pixel. Pixels with ≥50% forest cover were considered to indicate forest presence in our study. We estimated the forest cover size for each district by using the number of pixels defined as forest within each district. Deforestation was provided as a dichotomous variable based on whether there were tree-loss events during each year from 2001 to 2017. We assessed the deforestation level as the number of pixels indicating deforestation events within each district (Supplementary Figure S1), following a previous study [24]. To examine the possible long-term effects of deforestation, we calculated the 3-year cumulative deforestation level for each district as the main explanatory variable. 

The total population and number of farmers by year and district were obtained from the Korean Statistical Information Service (KOSIS) [20], to adjust the association between deforestation and scrub typhus incidence. Meteorological factors, including the annual average temperature, annual average relative humidity, total sunlight time, and total precipitation, were obtained from the Automatic Synoptic Observation System of Korea Meteorological Administration [21], which can be accessed from the website. Because the meteorological data were provided by each observation site with their location information, the data would not entirely represent each district. Therefore, ordinary kriging was used to generate interpolated values for the entire surface of Korea, and then average (temperature and relative humidity) or summed (sunlight time and precipitation) values were extracted for each district. Elevation data were obtained from Shuttle Radar Topography Mission data v4.1 [24], which provide 90-m-scale global elevation data in raster format, and we extracted the average altitudes of each district. The total area of each district was obtained from the KOSIS website [20].

The data described above were acquired for each year and district, and were modified to reflect the dynamic changes in administrative boundaries during the study period from 2006 to 2017. For example, three districts of Bucheon were united in 2016, whereas two new districts were introduced in Cheonan in 2008: Dongnam-gu and Seobuk-gu. For practical purposes, the different geographical classifications of administrative regions between years needed to be standardized, and we used the 2017 classification to this end. For instance, the populations in the three districts of Bucheon before 2016 were summed to give the population of Bucheon, and those of Dongnam-gu and Seobuk-gu in Cheonan before 2008 were estimated based on their area and that of Cheonan before 2008. 

### 2.2. Analysis

A descriptive analysis was performed to overview the differences between districts with higher and lower incidences of scrub typhus from 2006 to 2017, based on the median value. The means and standard deviations were provided. 

Although the covariates were selected based on the previous findings which reported associations with scrub typhus, not all covariates were included, and a variable selection process was implemented to avoid the issue of multi-collinearity. As previous studies suggested, variables with a variance inflation factor value greater than 10 [25] or one-to-one correlation coefficients greater than 0.8 were excluded.

Bayesian regression models using an Integrated Nested Laplace Approximation (INLA) approach [26] were used to examine the association between deforestation and scrub typhus. Assuming that the annual incidence of scrub typhus followed a Poisson distribution, we included a Poisson model. Negative binomial (NB), zero-inflated Poisson (ZIP) and zero-inflated negative binomial (ZINB) models were also employed to consider possible overdispersion, excessive zeros and both factors, respectively. Beside the four models, we additionally employed hierarchical Bayesian models [27] to reflect possible spatial autocorrelations in the residuals. These hierarchical models also comprised Poisson, NB, ZIP and ZINB models. Among the eight models, we determined the best fit model based on the deviance information criterion (DIC) [28]. The analyses were implemented using the INLA package [29,30] in R ver. 3.5.0 [31], and the results from all models were described with relative risks (RR) and 95% credible intervals (95% CI). 

We also conducted a sensitivity analysis according to the time period to define the deforestation level. Although we used the 3-year cumulative deforestation level as the main explanatory variable, the appropriateness of the 3-year period needs to be assessed. In this regard, we repeated the analysis using periods of 1 to 6 years, to examine the robustness of the study results.

## 3. Results

### 3.1. Descriptive Analysis

Table 2 presents the descriptive statistics for the 250 districts for the variables used in this study, stratified by incidence above and below the median. Deforestation level, number of farmers, annual mean temperature, precipitation, relative humidity, paddy field, and urban area land cover were higher in the high incidence districts (7.11 km^2^, 15,840, 13.40 °C, 1288 mm, 68.46%, 9700.58 m^2^, and 88.77 km^2^, respectively) than in the others.

### 3.2. The Association between Deforestation and Scrub Typhus

Table 3 and Table 4 show the results of the non-spatial and spatiotemporal models. In terms of the association between scrub typhus and deforestation level, the main explanatory variable in this study, all models showed significant positive associations, and the point estimates ranged between 1.15 and 1.22.

As the ZINB model that considered the spatial autocorrelation structure had the lowest DIC, we selected it as the best model. The RR of the interquartile range (IQR) increase in deforestation level was 1.20, and the 95% CI was 1.15–1.24. The annual mean temperature (RR = 2.28, 95% CI = 2.10–2.47), relative humidity (1.29, 1.21–1.38), amount of sunlight (1.18, 1.02–1.36), paddy field land cover (1.48, 1.37–1.59), and elevation (1.28, 1.19–1.38) showed significantly positive associations. Conversely, population density (RR = 0.70, 95% CI = 0.66–0.74), number of farmers (0.89, 0.81–0.99), budget dependency (0.81, 0.75–0.87), and forest land cover (0.75, 0.69–0.81) had significant negative associations.

Table 5 shows the results of the sensitivity analysis. All variables related to cumulative deforestation levels between 1 and 6 years in the past had significant associations, and the point estimates tended to increase with time up to 5 years.

## 4. Discussion

Using an ecological study design, this study examined the hypothesis that higher deforestation levels increase the risk of scrub typhus. All of the results, including the sensitivity analysis, showed that the incidence of scrub typhus tended to increase in the regions with greater deforestation. Several covariates showed significant associations in the final model, including sociodemographic, meteorological, and geographical variables.

The significant association between deforestation and scrub typhus was consistent with our hypothesis. The hypothesis was based on the supposed underlying mechanism that deforestation-induced secondary growth of scrub vegetation may increase the density of vectors. The finding is also consistent with studies of other vector-borne diseases [15,32]. Nevertheless, there could be other explanations related to the loss of diversity in wildlife communities from forest loss or habitat degradation [24]. First, a decrease in rodent diversity could increase the risk of scrub typhus by loss of the dilution effect [33], which is the theory that a higher host diversity (diversity of rodents in this case) decreases the risk of vector-borne diseases in humans by lowering the contact rate between competent natural hosts and infected vectors. Second, the decreased predator abundance or diversity induced by deforestation may suppress predatory pressures on rodents, although there is little evidence supporting this explanation. The association with deforestation seemed to be robust in this study, considering the results of the sensitivity analysis, which showed consistency among the models using different periods to assess the cumulative deforestation level. This also showed that the effects of deforestation would last up to 5 years, because the effect size increased with time up to 5 years.

The associations with other covariates were also consistent with published results. Annual mean temperature may increase the risk, possibly due to increasing mite activity [34], as reported previously [10]. Greater agricultural land use may increase the risk, since agricultural activities were risk factors, as previous studies have suggested [8,9]. Interestingly, the number of farmers was negatively associated in the final model, probably because the agricultural land use per farmer decreased as the number of farmers increased. However, the covariates should be interpreted cautiously because the variables included in the model were selected to adjust the association with deforestation. In other words, we may need to include other variables to adjust the effects of the covariates for proper interpretation.

The results implied that the issue of forest conservation is not limited to environmental sectors; public health authorities should also consider the problem. Although our results showed only the potential effect of deforestation on scrub typhus, other vector-borne diseases such as severe fever with thrombocytopenia syndrome, hemorrhagic fever with renal syndrome, and malaria are also likely associated with deforestation, because the underlying mechanism is applicable to these diseases. There is also evidence linking forest conservation to other public health benefits, such as improving mental health [35], climate change adaptation [36], and reducing the health effect of air pollution [37]. Therefore, public health authorities should be engaged in development issues that include deforestation and should evaluate their potential health impacts. This is an example of the One Health approach, which was defined as “the collaborative effort of multiple disciplines—working locally, nationally and globally—to attain the optimal health of people, animals, and our environment” by the American Veterinary Medical Association [38].

This study has several limitations. First, it did not include interventions by health authorities, such as education programs and public campaigns, due to a lack of data. Although adding these variables could help determine the effects of the interventions, it would not affect the association between deforestation and the incidence of scrub typhus, because an association between deforestation and the intervention is unlikely. Second, the measurements of deforestation might not be accurate because they were estimated from remote-sensing satellite images. However, the data showed reliability as they were consistent with other data [19] and various studies have used such data [24,39,40], indicating the acceptable validity of the data. Third, the modifiable areal unit problem [41] would produce bias in the results, as we used aggregated data throughout the study. In other words, areal classifications other than the administrative boundaries used in this study, such as rectangular grids with 1 km^2^ resolution, could give different results. Because the reported number of scrub typhus cases could only be obtained from each district, follow-up studies with more sophisticated designs are needed to examine whether the results can be replicated. Fourth, the spatial distribution of scrub typhus was based on the registered residential address of the patient which would not represent where their infection was acquired. While the potential bias would be practically inevitable, follow-up studies will be needed to examine the replicability of the results. Fifth, some false negative scrub typhus cases would be included in the analysis because we used not only confirmed cases but also suspected cases to measure scrub typhus incidence. While it was inevitable since KCDC did not provide the specific number of confirmed cases by district level, including suspected cases may be beneficial to offset the false negatives by the laboratory tests. For example, although the IFA test shows high sensitivity, it is costly and needs well trained experts [42]. Consequently, the sensitive test is likely less available in the rural areas with higher incidence [43]. Nonetheless, interpretation of the study results with the current case definition should be cautious, and studies using other case definitions (e.g., confirmed only or categorized by laboratory test methods) should be followed.

Our findings showed that integrating environmental factors as a public health issue is important for understanding the dynamics of infectious diseases [44], and health authorities should be more engaged in conservation issues. Although we showed the association between deforestation and scrub typhus incidence in South Korea, further studies would be needed for generality of the study results in other countries. That is because disease ecology varies by different regions. For example, one of the vectors in Japan is Leptotrombidium akamushi which is mainly distributed in a basin of a river, not in bush or scrub vegetation as Leptotrombidium pallidum and Leptotrombidium scutellare are distributed in Korea.

## 5. Conclusions

In this study, we investigated the association between deforestation and scrub typhus incidence in South Korea by using an ecological study design. The results showed that districts with higher deforestation tended to have significantly higher incidence rates. Although the interpretation should be cautious due to several study limitations, the potential positive association provides a novel perspective for scrub typhus control and prevention. Follow-up studies are recommended to examine the replicability of the study results not only in South Korea, but also in other endemic countries. 

## Figures and Tables

**Table 1:** Data acquired in this study.

Category 1	Category 2	Variables	Data Source	Reference
Response variable	Outcome	Scrub typhus incidence	KCDC ^a^	[2]
Explanatory variable	Deforestation	Deforestation	GFC ^b^	[19]
Covariates	Sociodemographics	Population density of	KOSIS ^c^	[20]
		farmers	KOSIS	
	Economy	Budget dependency	KOSIS	
	Meteorology	Average temperature	KMA ASOS ^d^	[21]
		Precipitation	KMA ASOS	
		Relative humidity	KMA ASOS	
		Total sunlight time	KMA ASOS	
	Land cover	Agriculture (paddy)	KOSIS	
		Agriculture (all)	KOSIS	
		Urban area	KOSIS	
		Forest	GFC	
	Geography	Elevation	SRTM ^e^	[22]
		Extent	KOSIS	

^a^ KCDC: Korea Centers for Disease Control and Prevention, ^b^ GFC: Global Forest Change, ^c^ KOSIS: Korean Statistical Information Service, ^d^ KMA ASOS: Korea Meteorological Administration Automatic Synoptic Observation System, ^e^ SRTM: Shuttle Radar Topography Mission. *Note:* The total extent area and agricultural land cover (all) were excluded from the model because the variance inflation factor was greater than 10.

**Table 2:** Descriptive analysis of the variables used in the models by the incidence of scrub typhus.

Variables	Districts with Higher Incidence (>Median ^a^, n = 125) Mean (±SD)	Districts with Lower Incidence (≤Median ^a^, n = 125) Mean (±SD)
Scrub typhus cases (all cases; 2006–2017)	628.22 ± 314.8	97.98 ± 59.7
Deforestation (2006–2017, sum, km^2^)	7.11 ± 9.5	6.45 ± 11.0
Population density (10^3^ per km^2^)	1.87 ± 3.5	6.19 ± 7.5
Farmers (10^3^)	15.84 ± 10.9	7.43 ± 8.0
Budget dependency (%)	24.09 ± 12.7	34.92 ± 17.6
Mean temperature (°C)	13.40 ± 0.9	12.20 ± 0.8
Precipitation (mm)	1288.17 ± 133.4	1274.31 ± 104.4
Relative humidity (%)	68.46 ± 3.4	66.98 ± 1.4
Total sunlight time (h)	2132.03 ± 40.3	2157.14 ± 21.1
Agriculture (paddy, m^2^)	9700.58 ± 8158.0	3875.44 ± 5150.8
Urban area (km^2^)	88.77 ± 87.4	55.52 ± 56.6
Forest (km^2^)	198.07 ± 194.0	222.24 ± 354.1
Elevation (mean, m)	158.33 ± 117.0	182.41 ± 175.8

*Note*: All variables in this table were used in the regression models in this study. ^a^ Among the 250 districts, the median incidence of scrub typhus was 263.5 cases during the period 2006–2017.

**Table 3:** Relative risks of scrub typhus by interquartile range increase for each explanatory variable from the non-spatial models.

Variables	Relative Risk (95% Credible Interval)			
	Poisson	ZIP ^a^	NB ^b^	ZINB ^c^
Deforestation	1.20 (1.20–1.21)	1.19 (1.19–1.20)	1.22 (1.17–1.27)	1.22 (1.18–1.26)
Population density	0.57 (0.57–0.58)	0.56 (0.55–0.57)	0.67 (0.64–0.70)	0.66 (0.63–0.69)
Farmers	0.76 (0.75–0.77)	0.73 (0.72–0.74)	0.94 (0.84–1.04)	0.93 (0.84–1.03)
Budget dependency	0.86 (0.85–0.87)	0.90 (0.89–0.92)	0.77 (0.72–0.82)	0.79 (0.74–0.84)
Mean temperature	1.97 (1.95–2.00)	1.98 (1.96–2.01)	2.43 (2.27–2.61)	2.47 (2.30–2.64)
Precipitation	1.02 (1.00–1.03)	1.02 (1.00–1.03)	0.97 (0.90–1.04)	0.97 (0.90–1.03)
Relative humidity	1.15 (1.14–1.16)	1.16 (1.15–1.17)	1.32 (1.25–1.40)	1.32 (1.25–1.40)
Total sunlight time	1.04 (1.01–1.08)	1.05 (1.02 -1.08)	1.10 (0.96–1.26)	1.11 (0.97–1.26)
Agriculture	1.51 (1.50–1.53)	1.50 (1.49–1.52)	1.46 (1.35–1.58)	1.44 (1.34–1.56)
Urban area	0.98 (0.98–0.99)	0.97 (0.97–0.98)	0.99 (0.96–1.02)	0.99 (0.96–1.02)
Forest	0.72 (0.71–0.74)	0.74 (0.73–0.76)	0.67 (0.62–0.73)	0.68 (0.63–0.73)
Elevation	1.27 (1.25–1.29)	1.30 (1.28–1.32)	1.30 (1.21–1.41)	1.31 (1.21–1.41)
DIC ^d^	65,791.8	62,488.92	24,522.27	24,498.22

*Note*: Bayesian regression models with Integrated Nested Laplace Approximation (INLA) were used and the time variable (year) was included as a random walk structure, but spatial structure was not considered in the model. ^a^ Zero-inflated Poisson; ^b^ Negative binomial; ^c^ Zero-inflated negative binomial; ^d^ Deviance information criterion.

**Table 4:** Relative risks of scrub typhus by interquartile range increase for each explanatory variable from spatiotemporal models.

Variables	Relative risk (95% Credible Interval)			
	Poisson	ZIP ^a^	NB ^b^	ZINB ^c^
Deforestation	1.16 (1.15–1.17)	1.15 (1.14–1.16)	1.20 (1.15–1.25)	1.20 (1.15–1.24)
Population density	0.63 (0.62–0.65)	0.64 (0.63–0.65)	0.70 (0.67–0.74)	0.70 (0.66–0.74)
Farmers	0.86 (0.84–0.88)	0.83 (0.81–0.84)	0.92 (0.83–1.02)	0.89 (0.81–0.99)
Budget dependency	0.88 (0.87–0.90)	0.93 (0.91–0.95)	0.77 (0.71–0.83)	0.81 (0.75–0.87)
Mean temperature	1.65 (1.62–1.69)	1.69 (1.66–1.73)	2.24 (2.06–2.43)	2.28 (2.10–2.47)
Precipitation	0.93 (0.91–0.95)	0.92 (0.90–0.94)	0.94 (0.86–1.02)	0.92 (0.85–1.00)
Relative humidity	1.09 (1.07–1.11)	1.10 (1.09–1.12)	1.29 (1.20–1.38)	1.29 (1.21–1.38)
Total sunlight time	1.20 (1.14–1.25)	1.16 (1.11–1.22)	1.17 (1.02–1.36)	1.18 (1.02–1.36)
Agriculture	1.41 (1.39–1.42)	1.40 (1.39–1.42)	1.50 (1.39–1.62)	1.48 (1.37–1.59)
Urban area	0.97 (0.82–0.86)	0.97 (0.96–0.97)	1.00 (0.97–1.04)	1.00 (0.97–1.03)
Forest	0.84 (0.82–0.86)	0.88 (0.86–0.89)	0.73 (0.67–0.79)	0.75 (0.69–0.81)
Elevation	1.33 (1.30–1.35)	1.31 (1.28–1.33)	1.29 (1.20–1.39)	1.28 (1.19–1.38)
DIC ^d^	52,380.41	49,848.48	24,187.01	24,109.29

*Note*: Bayesian regression models with INLA were used and the time variable (year) was included as a random walk structure, and spatial autocorrelation was also considered with bym models. ^a^ Zero-inflated Poisson; ^b^ Negative binomial; ^c^ Zero-inflated negative binomial; ^d^ Deviance information criterion.

**Table 5:** The results of the sensitivity analysis using different deforestation accumulation periods.

Accumulation Period for Assessing Deforestation	Relative Risk (95% Credible Interval)	Deviance Information Criterion
One years	1.153 (1.116–1.192)	24,123.36
Two years	1.188 (1.146–1.233)	24,112.64
Three years	1.196 (1.152–1.242)	24,109.29
Four years	1.207 (1.161–1.256)	24,111.03
Five years	1.213 (1.167–1.261)	24,101.32
Six years	1.207 (1.162–1.255)	24,102.20

*Note:* The relative risks (RR) were for an increase in deforestation level by interquartile range, and the zero-inflated negative binomial model with spatial autocorrelation, which had the lowest deviance information criterion value, was used to produce the RR. Other variables were used to adjust for potential confounding effects, including population density, the number of farmers, budget dependency, temperature, precipitation, relative humidity, amount of sunlight, agricultural land use, urban land cover, forest land cover, and altitude.

## Supplementary Materials

The following are available online at https://www.mdpi.com/1660-4601/16/9/1518/s1, Figure S1: Data acquisition and preprocessing for forest cover and deforestation.

## References

[1] Lee H.W., Cho P.Y., Moon S.U., Na B.K., Kang Y.J., Sohn Y., Youn S.K., Hong Y., Kim T.S. (2015). Current situation of scrub typhus in South Korea from 2001–2013. Parasit. Vectors.

[2] Korea Center for Disease Control and Prevention Infectious Disease Portal. http://www.cdc.go.kr/npt/.

[3] Korea Center for Disease Control and Prevention (2009). Determination of vector species of tsutsugamushi disease and their geographical distribution in Korea. Public Health Weekly Rep..

[4] Park S.W., Ha N.Y., Ryu B., Bang J.H., Song H., Kim Y., Kim G., Oh M.D., Cho N.H., Lee J.K. (2015). Urbanization of scrub typhus disease in South Korea. PLoS Negl. Trop. Dis..

[5] Min K.D., Cho S.I. (2018). Mathematical modeling for scrub typhus and its implications for disease control. J. Korean Med. Sci..

[6] Trowbridge P., P D., Premkumar P.S., Varghese G.M. (2017). Prevalence and risk factors for scrub typhus in south India. Trop. Med. Int. Health.

[7] Ogawa M., Hagiwara T., Kishimoto T., Shiga S., Yoshida Y., Furuya Y., Kaiho I., Ito T., Nemoto H., Yamamoto N. (2002). Scrub typhus in Japan: Epidemiology and clinical features of cases reported in 1998. Am. J. Trop. Med. Hyg..

[8] Kim D.S., Acharya D., Lee K., Yoo S.J., Park J.H., Lim H.S. (2018). Awareness and Work-Related Factors Associated with Scrub Typhus: A Case-Control Study from South Korea. Int. J. Environ. Res. Public Health.

[9] Ma C.J., Oh G.J., Kang G.U., Lee J.M., Lee D.U., Nam H.S., Ryu S.Y., Lee Y.H. (2017). Differences in agricultural activities related to incidence of scrub typhus between Korea and Japan. Epidemiol. Health.

[10] Li T., Yang Z., Dong Z., Wang M. (2014). Meteorological factors and risk of scrub typhus in Guangzhou, southern China, 2006–2012. BMC Infect. Dis..

[11] Kwak J., Kim S., Kim G., Singh V.P., Hong S., Kim H.S. (2015). Scrub Typhus Incidence Modeling with Meteorological Factors in South Korea. Int. J. Environ. Res. Public Health.

[12] Wei Y., Huang Y., Li X., Ma Y., Tao X., Wu X., Yang Z. (2017). Climate variability, animal reservoir and transmission of scrub typhus in Southern China. PLoS Negl. Trop. Dis..

[13] Jeung Y.S., Kim C.M., Yun N.R., Kim S.W., Han M.A., Kim D.M. (2016). Effect of Latitude and Seasonal Variation on Scrub Typhus, South Korea, 2001–2013. Am. J. Trop. Med. Hyg..

[14] Walker D.H. (2016). Scrub typhus—Scientific neglect, ever-widening impact. N. Engl. J. Med..

[15] Fornace K.M., Abidin T.R., Alexander N., Brock P., Grigg M.J., Murphy A., William T., Menon J., Drakeley C.J., Cox J. (2016). Association between landscape factors and spatial patterns of Plasmodium knowlesi infections in Sabah, Malaysia. Emerg. Infect. Dis..

[16] Terrazas W.C.M., Sampaio V.D.S., De Castro D.B., Pinto R.C., De Albuquerque B.C., Sadahiro M., Dos Passos R.A., Braga J.U. (2015). Deforestation, drainage network, indigenous status, and geographical differences of malaria in the state of Amazonas. Mala J..

[17] Hahn M.B., Gangnon R.E., Barcellos C., Asner G.P., Patz J.A. (2014). Influence of deforestation, logging, and fire on malaria in the Brazilian Amazon. PLoS ONE.

[18] Wayant N.M., Maldonado D., de Arias A.R., Cousiño B., Goodin D.G. (2010). Correlation between normalized difference vegetation index and malaria in a subtropical rain forest undergoing rapid anthropogenic alteration. Geospatial Health.

[19] Hansen M.C., Potapov P.V., Moore R., Hancher M., Turubanova S.A., Tyukavina A., Thau D., Stehman S.V., Goetz S.J., Loveland T.R. (2013). High-resolution global maps of 21st-century forest cover change. Science.

[20] Korean Statistical Information Service. http://kosis.kr/index/index.do.

[21] Korea Meteorological Administration Automatic Synoptic Observation System. https://data.kma.go.kr/data/grnd/selectAsosRltmList.do?pgmNo=36.

[22] Jarvis A., Reuter H., Nelson A., Guevara E. Hole-Filled SRTM for the globe Version 4. http://srtm.csi.cgiar.org.

[23] Korea Center for Disease Control and Prevention (2017). Case Definitions for National Notifiable Infectious Diseases.

[24] Betts M.G., Wolf C., Ripple W.J., Phalan B., Millers K.A., Duarte A., Butchart S.H.M., Levi T. (2017). Global forest loss disproportionately erodes biodiversity in intact landscapes. Nature.

[25] Lin F.J. (2008). Solving multicollinearity in the process of fitting regression model using the nested estimate procedure. Qual Quant..

[26] Blangiardo M., Cameletti M., Baio G., Rue H. (2013). Spatial and spatio-temporal models with R-INLA. Spat. Spatiotemporal. Epidemiol..

[27] Beguin J., Martino S., Rue H., Cumming S.G. (2012). Hierarchical analysis of spatially autocorrelated ecological data using integrated nested laplace approximation. Methods Ecol. Evol..

[28] Spiegelhalter D.J., Best N.G., Carlin B.P., Van Der Linde A. (2002). Bayesian measures of model complexity and fit. J. R. Stat. Soc. Ser. B Stat. Methodol..

[29] Martino S., Rue H. (2009). Implementing Approximate Bayesian Inference Using Integrated Nested Laplace Approximation: A Manual for the Inla Program.

[30] Rue H., Martino S., Chopin N. (2009). Approximate bayesian inference for latent gaussian models by using integrated nested laplace approximations. J. R Stat. Soc. Ser. B Stat. Methodol..

[31] R Core Team (2018). R: A Language and Environment for Statistical Computing.

[32] Valle D., Tucker Lima J.M. (2014). Large-scale drivers of malaria and priority areas for prevention and control in the brazilian amazon region using a novel multi-pathogen geospatial model. Mala J..

[33] Ostfeld R.S., Keesing F. (2012). Effects of host diversity on infectious disease. Annu. Rev. Ecol. Evol. Syst..

[34] Van Peenen P.F., Lien J.C., Santana F.J., See R. (1976). Correlation of chigger abundance with temperature at a hyperendemic focus of scrub typhus. J. Parasitol..

[35] Min K.B., Kim H.J., Kim H.J., Min J.Y. (2017). Parks and green areas and the risk for depression and suicidal indicators. Int. J. Public Health.

[36] D’Amato G., Vitale C., Rosario N., Neto H.J.C., Chong-Silva D.C., Mendonca F., Perini J., Landgraf L., Sole D., Sanchez-Borges M. (2017). Climate change, allergy and asthma, and the role of tropical forests. World Allergy Organ. J..

[37] Nowak D.J., Hirabayashi S., Bodine A., Greenfield E. (2014). Tree and forest effects on air quality and human health in the united states. Environ. Pollut..

[38] One Health Initiative Task Force (2008). One Health: A New Professional Imperative.

[39] Morris A.L., Guégan J.-F., Andreou D., Marsollier L., Carolan K., Le Croller M., Sanhueza D., Gozlan R.E. (2016). Deforestation-driven food-web collapse linked to emerging tropical infectious disease, mycobacterium ulcerans. Sci. Adv..

[40] Rulli M.C., Santini M., Hayman D.T., D’Odorico P. (2017). The nexus between forest fragmentation in africa and ebola virus disease outbreaks. Sci. Rep..

[41] Openshaw S. (1984). The Modifiable Areal Unit Problem.

[42] Jiang J., Marienau K.J., May L.A., Beecham H.J., Wilkinson R., Ching W.M., Richards A.L. (2003). Laboratory diagnosis of two scrub typhus outbreaks at Camp Fuji, Japan in 2000 and 2001 by enzyme-linked immunosorbent assay, rapid flow assay, and Western blot assay using outer membrane 56-kD recombinant proteins. Am. J. Trop. Med. Hyg..

[43] Kim Y.J., Yeo S.J., Park S.J., Woo Y.J., Kim M.W., Kim S.H., Chang I., Jeon S.H., Park B.J., Song G.J. (2013). Improvement of the diagnostic sensitivity of scrub typhus using a mixture of recombinant antigens derived from Orientia tsutsugamushi serotypes. J. Korean Med. Sci..

[44] Ryu S., Kim B.I., Lim J.S., Tan C.S., Chun B.C. (2017). One health perspectives on emerging public health threats. J. Prev. Med. Public Health.

