# Supplementary figures and images for: Deforestation Increases the Risk of Scrub Typhus in Korea
Source: Int J Environ Res Public Health. 2019 Apr 29;16(9):1518. doi: 10.3390/ijerph16091518 (PMC6539434; doi:10.3390/ijerph16091518)

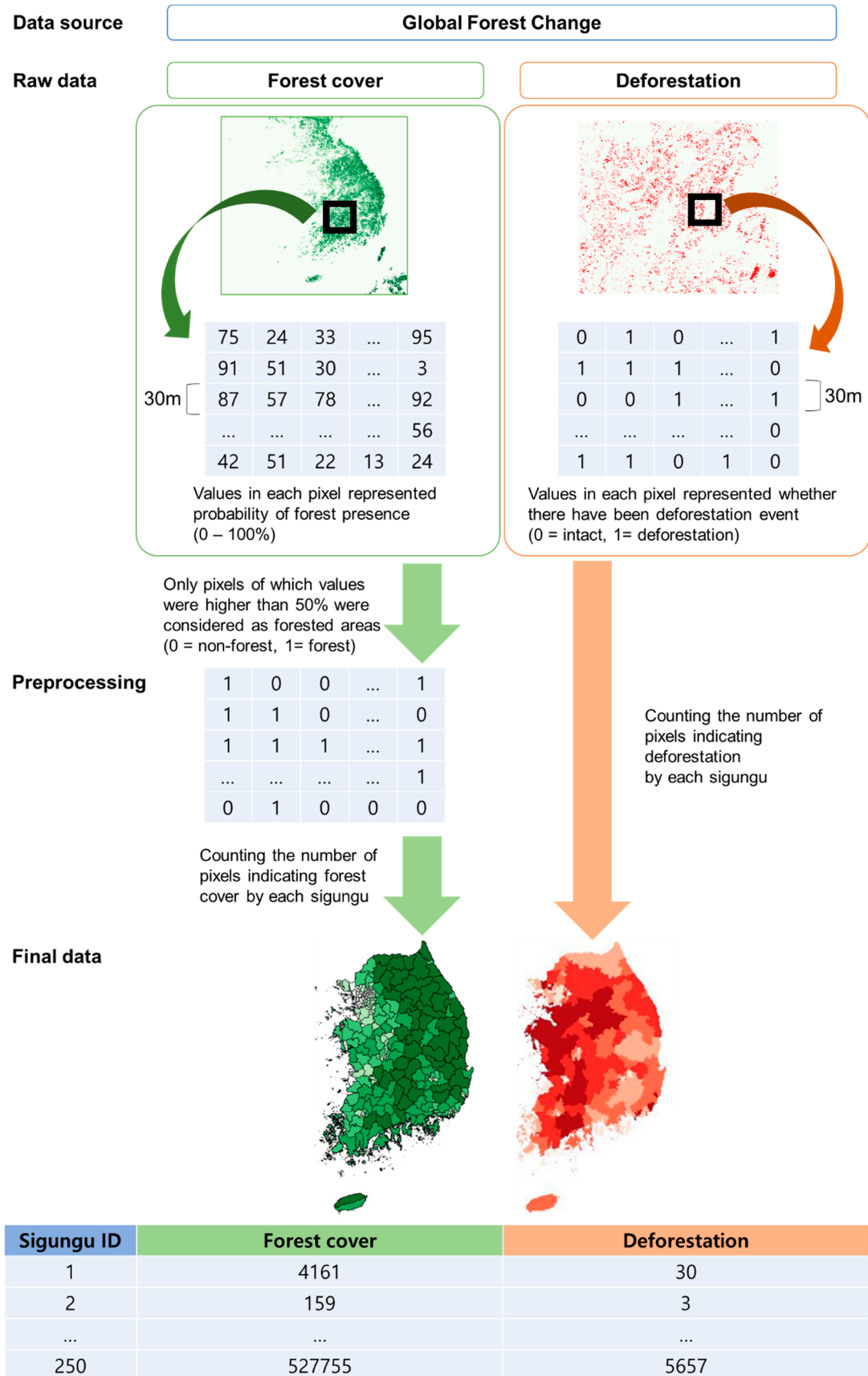

**Figure S1.** Data acquisition and preprocessing for forest cover and deforestation

Supplement: Supplementary file 1 [file ijerph-16-01518-s001.pdf]
